# Occupational Factors on QOL of University Teachers

**DOI:** 10.3390/ijerph22101546

**Published:** 2025-10-11

**Authors:** Flavio Henrique Rodrigues da Silva, Maria Alves Barbosa, Celmo Celeno Porto, Eliane Gouveia de Morais Sanchez, Luiz Almeida da Silva, Ludmila Grego Maia, Marianne Lucena da Silva, Hugo Machado Sanchez

**Affiliations:** 1 Postgraduate Program in Health Sciences, Faculty of Medicine, Federal University of Goias (UFG), Goiânia 74690-900, Brazil; flaviohrsmk@gmail.com (F.H.R.d.S.); maria.malves@gmail.com (M.A.B.); celmo1934@gmail.com (C.C.P.); 2 Department of Physiotherapy, Institute of Health Sciences, Federal University of Jataí (UFJ), Jataí 75801-615, Brazil; egmfisio@ufj.edu.br; 3 Department of Nursing, Institute of Health Sciences, Federal University of Catalão (UFCAT), Catalão 75705-220, Brazil; enferluiz@yahoo.com.br; 4 Department of Nursing, Institute of Health Sciences, Federal University of Jataí (UFJ), Jataí 75801-615, Brazil; ludmila@ufj.edu.br; 5 Institute of Public Health Sciences, Federal University of Brasilia (UnB), Brasilia 70910-900, Brazil; mariannebsb@gmail.com

**Keywords:** work, professor, teacher, health, evaluation

## Abstract

This study aimed to analyze which work-related factors may influence the quality of life (QOL) and quality of work life (QWL) of academic teachers from different fields of knowledge, as well as to verify the correlation between QOL and QWL. It is a cross-sectional study in which data were collected using a sociodemographic questionnaire containing work-related questions, the WHOQOL-BREF, and the TQWL-42 instruments. The sample consisted of 284 academic teachers from various disciplines. The total population at the higher education institution (HEI) comprised 386 faculty members, and the sample size was determined using OpenEpi^®^, with a 95% confidence level. The results showed no significant differences in QOL and QWL between the different fields of knowledge. However, both QOL and QWL were influenced by several work-related factors, including higher remuneration, holding a statutory employment position, not needing to relocate from one’s home city to work as a professor, adequate lighting, comfortable room temperature, lower noise levels, sufficient material resources, and smaller class sizes. Additionally, a positive correlation between QOL and QWL was observed. In conclusion, both QOL and QWL are influenced by organizational and work-related conditions associated with the academic profession, rather than by disciplinary areas. These findings suggest that the work environment and personal life of academic staff are interdependent, and efforts to improve one may positively impact the other.

## 1. Introduction

The concept of quality of life (QOL) is broad and multifaceted, resulting from the interaction of biological, psychological, and social factors that affect individuals within their societal context. It is also dynamic, varying according to the socio-cultural environment in which an individual is situated [1]. According to the World Health Organization (WHO) QOL Group, QOL is defined as an individual’s perception of their position in life, within the context of the culture and value systems in which they live and in relation to their goals, expectations, standards, and concerns [2].

An individual’s QOL is intrinsically linked to job satisfaction and other variables associated with occupational activity. These factors collectively determine the quality of work life (QWL) [3]. QWL refers to an individual’s perception of their working conditions, emphasizing subjective elements such as satisfaction with the workplace, physical environment, availability of material resources, salary, organizational structure, health, and occupational safety [4]. It also encompasses personal and social expectations, pride and pleasure in one’s work, physical and emotional well-being, self-esteem, institutional reputation, career development opportunities, and the protection of workers’ rights [5,6].

Teachers hold a vital position in society, as they are central to intellectual development and the formation of social relationships. Through theoretical and practical and intellectual and administrative activities, teachers foster autonomy, critical thinking, and responsibility among students [5,6,7]. However, teaching is recognized as a profession that imposes considerable emotional and cognitive demands. Educators frequently engage with heterogeneous student populations, manage high student turnover, face continuous pressure for academic qualification and scientific advancement, fulfill multiple institutional responsibilities, and undertake a substantial volume of extracurricular work [5,6,7].

A variety of factors inherent to teaching can negatively affect both QOL and QWL. These include inadequate or deficient infrastructure, insufficient teaching resources, excessive bureaucratic duties, inefficient administrative procedures, low remuneration, long working hours, lack of professional recognition and appreciation, limited career development, and absence of structured career planning [7,8,9,10].

In the study by Molinero, Teles, and Cotrin, which was conducted during the pandemic, when teachers taught classes digitally and teleworking was widely used, they found that teleworking, noise, temperature, lighting, physical resources, and technological difficulties negatively impacted teachers’ QWL [11].

The accumulation of teaching, administrative, research, and outreach responsibilities often necessitates the completion of work-related tasks outside of formal working hours, frequently at home. This significantly reduces time available for personal activities, rest, and leisure [9]. Furthermore, technological advancements have blurred the boundaries between professional and personal life, allowing work to occur from virtually any location. While this can offer flexibility, it often contributes to an increased workload and heightens health risks among educators [7,12].

Given that higher education plays a fundamental role in national development, enhancing the QWL of academic staff emerges as a critical strategy. This involves fostering healthier and more stimulating work environments, minimizing occupational risk factors, and promoting a balance between professional and personal life [5,6]. Despite the significance of the teaching profession, limited research has explored the occupational factors influencing QOL and QWL across various academic disciplines.

Therefore, the objective of this study was to analyze the work-related factors that may influence the QOL and QWL of professors from different fields of knowledge and to investigate the association between QOL and QWL.

## 2. Materials and Methods

This is a cross-sectional, descriptive, observational study conducted at a public (government-funded) higher education institution (HEI) located in a state in the Central-West region of Brazil. The HEI employs both statutory and non-statutory faculty, with non-statutory teachers contracted based on the number of classes taught.

The study was approved by the Research Ethics Committee of the University of Rio Verde (Opinion No. 042821). Written informed consent was obtained from all participants prior to inclusion.

### 2.1. Sampling and Participants

A stratified random probability sampling method was used, with stratification based on academic field, sex, and age. Sample size calculations were performed separately for each combination of these variables using OpenEpi^®^ with a 95% confidence level. From a total population of 386 faculty members, the minimum representative sample was determined to be 193 participants. Although all eligible faculty members were invited to participate, 284 valid questionnaires were ultimately returned, excluding those who met the exclusion criteria.

Inclusion criteria consisted of faculty members with more than six months of teaching experience, regardless of employment type. Exclusion criteria included incomplete questionnaires, faculty members engaged exclusively in administrative roles, those on leave during the data collection period, and faculty enrolled in Stricto Sensu postgraduate programs during their teaching practicum. If any of the three instruments were incompletely filled out, all data from that respondent were excluded.

### 2.2. Data Collection Instruments

Data collection was conducted simultaneously across all academic programs at the HEI using three instruments:

1. A structured sociodemographic questionnaire, designed by the researchers, containing categorical variables. This instrument underwent content validation by three experts in the field and methodology. Following this, necessary revisions were made, and two pre-tests were conducted. Feedback from the pre-tests was used to finalize the instrument.
2. The Total Quality of Work Life (TQWL-42) instrument, developed and validated by Pedroso et al. [13], assesses QWL across various populations. Based on classical QWL models and the WHOQOL-100 framework, it includes 42 Likert-scale items grouped into five domains:
  - Sphere 1: Biological/Physiological
  - Sphere 2: Psychological/Behavioral
  - Sphere 3: Sociological/Relational
  - Sphere 4: Economic/Political
  - Sphere 5: Environmental/Organizational
3. The WHOQOL-BREF, developed by the WHO in 1998 and validated in Portuguese by Fleck et al. [14], includes 26 Likert-scale items divided into four domains:
  - Domain 1: Physical
  - Domain 2: Psychological
  - Domain 3: Social Relationships
  - Domain 4: Environmental

The Likert scales in both instruments range from 0 to 5. The maximum possible score is 20 points for the WHOQOL-BREF and 5 points for TQWL-42.

All questionnaires were self-administered, although the research team was available to provide clarification if needed, making the process semi-assisted when necessary.

### 2.3. Instrument Reliability

To verify internal consistency in this population, Cronbach’s Alpha was calculated. The TQWL-42 instrument showed a mean of α = 0.80, with the following domain-specific values:

- Biological/Physiological: α = 0.78
- Psychological/Behavioral: α = 0.84
- Sociological/Relational: α = 0.78
- Economic/Political: α = 0.81
- Environmental/Organizational: α = 0.79

The WHOQOL-BREF instrument demonstrated a mean of α = 0.85:

- Physical domain: α = 0.90
- Psychological domain: α = 0.86
- Social Relationships domain: α = 0.83
- Environmental domain: α = 0.81

### 2.4. Data Analysis

Data were initially organized in Microsoft Excel^®^ and subsequently analyzed using SPSS^®^ version 20.0. Descriptive statistics were calculated, followed by the Shapiro–Wilk test to assess normality.

To compare QOL domain scores and QWL sphere scores, the Kruskal–Wallis test was used, both for the overall sample and for groups stratified by academic discipline.

To assess associations between independent variables and both QOL and QWL scores, the following was performed:

- Mann–Whitney U test was applied for variables with two groups.
- Kruskal–Wallis test was applied for variables with more than two groups.

Spearman’s correlation coefficient was used to assess relationships between sociodemographic variables and scores from the WHOQOL-BREF and TQWL-42, as well as the correlation between QOL and QWL.

Additionally, simple linear regression was performed to evaluate the association between the following:

- Administrative workload and QOL.
- Number of students in practical classes and QWL.

Statistical significance was set at *p* < 0.05.

## 3. Results

The sample of this present study consisted of 284 teachers; such a sample was chosen after excluding up to 11 questionnaires for inadequate filling and 29 due to the framework of exclusion. At the mean, ages were as follows: agricultural: 41.29 ± 9.23, health: 39.21 ± 7.75, exact: 37 ± 11.22, and human: 39.38 ± 10.39.

The QOL analysis of teachers showed an overall score of 15.20 ± 1.74 and 3.42 ± 0.41 for QWL (Table 1). These scores represent intermediate satisfaction with QOL and QWL by teachers. As shown in Table 1, the physical, psychological, and social relations domains obtained higher scores compared with the environmental score. Furthermore, without reference to the QWL, the economic–political sphere obtained the lowest score.

In Table 1, the domains analyzed by the QOL questionnaire and spheres of the QWL questionnaire are shown. In the comparative analysis between the domains of QOL, it can be seen that the environmental domain score was significantly lower (*p* = 0.000) when compared with physical, psychological, and social relations domains. Regarding the spheres that comprise the QWL, it was found that the score of the economic/political sphere was significantly lower (*p* = 0.000) than the score of the other spheres, as well as the score for biological/physiological sphere was lower when compared with psychological/behavioral (*p* = 0.005) and sociological/relational ones (*p* = 0.000). Moreover, it was found that the score of the psychological/behavioral sphere was statistically higher (*p* = 0.000) than those obtained in the sociological/relational and environmental/organizational spheres.

In Table 2, sociodemographic data were categorized; QOL and QWL spread over the area of knowledge. When comparing, through the Mann–Whitney U test, the QOL between sexes, there was no difference, however when comparing the QWL, it was found that men (QWL = 3.47 ± 0.4) reported a significantly higher QWL (*p* = 0.025), if compared with women (QWL = 3.35 ± 0.42) (Table 2).

Table 2 shows the values of the QOL and QWL comparisons for each variable related to work. In the income variable, it was observed that teachers with incomes above 10 min wages (QOL = 15.7 ± 2.03) had a significantly better QOL (*p* = 0.017) if compared with those whose income was of 2–3 minimum wages (QOL = 14.77 ± 1.48). Regarding QWL, it was found that teachers with incomes of 3–5 wages (QWL = 3.57 ± 0.43) had a significantly better QWL (*p* = 0.015) when compared with teachers earning 2–3 minimum wages (QWL = 3.26 ± 0.39).

The same table also shows the significant difference (*p* = 0.005) in the perception of QOL among teachers with statutory regulations at work (QOL = 16.51 ± 1.29) and teachers with an employment contract (QOL = 15.05 ± 1.64). Regarding QWL, teachers who did not have to drive away from their home city to work as teachers (QWL = 3.43 ± 0.42) reported a statistically better perception (*p* = 0.049) compared with the ones who had to travel long distances (QWL = 3.27 ± 0.41). Other analyses of sociodemographic data relayed in Table 2 were not relevant.

Comparing areas of QOL and QWL, using Kruskal–Wallis, there was no difference; women had *p* = 0.843 in the comparison of QOL and *p* = 0.198 in that of QWL.

Table 3 shows mean values and comparison of scores of the domains and spheres in each area of knowledge. In comparisons among domains that form the QOL, it was found that, in all areas, the physical domain was statistically higher (*p* = 0.004) than the environmental domain. Comparing the social relations domain and environmental one, it was found that the social relations domain was statistically higher (*p* = 0.004) in areas of health, human, and agriculture, while in the comparison between social affairs and psychological domains, this last one was significantly higher (*p* = 0.002) in the health/human/agricultural areas (Table 3).

Regarding QWL, it was found that the scores of the psychological/behavioral sphere was statistically higher (*p* = 0.004) than those obtained in the biological/physiological and economic/political spheres in all areas. The sociological/relational sphere was significantly higher (*p* = 0.031) than the biological/physiological one in health; and for the biological/physiological sphere, it was significantly higher (*p* = 0.027) than the economic/policy in exact sciences. Scores of the psychological/behavioral sphere were higher statistically (*p* = 0.004) than the sociological/relational one, in the health/human/agricultural areas; and were also higher than the environmental/organizational sphere (*p* = 0.005) in the health/human/exact areas. Finally, the economic/political sphere was statistically lower compared with the sociological/relational (*p* = 0.010) in the health/human/exact areas, and also lower than the environmental/organizational sphere in the health/human/agricultural ones (Table 3).

In Table 4, the categorized data related to work QOL and QWL are presented, spread over the area of knowledge. As for the work environment, it was found that teachers who worked in environments with better lighting (QWL = 3.48 ± 0.41) reported a statistically better QWL (*p* = 0.000) than those who worked in environments with regular lighting (QWL = 3.32 ± 0.41) or insufficient lighting (QWL = 3.03 ± 0.35). Regarding the influence of temperature on QWL, it was noted that teachers who worked in environments that provided greater thermal comfort (QWL = 3.50 ± 0.4), showed a significantly better QWL (*p* = 0.001) than those who informed working in an uncomfortable work environment (QWL = 3.31 ± 0.4). As for the noise in the teaching activity, both QOL and QWL were influenced, such that in QOL, teachers who worked with little noise interference (QOL = 15.46 ± 1.7) reported a significantly better QOL (*p* = 0.048) than those who worked with moderate noise (QOL = 14.92 ± 1.7), while in QWL, it was found that workers who worked without noise interference (QWL = 3.71 ± 0.39) or with little noise (QWL = 3.52 ± 0.41) demonstrated a significantly better QWL (*p* = 0.000) compared with those who reported working with moderate noise (QWL = 3.34 ± 0.4) or excessive noise (QWL = 3.3 ± 0.4) (Table 4).

Another factor that impacted QOL and QWL was resources for teaching, in this case those who realized ideal resources for activities (QOL = 15.67 ± 1.58) showed a statistically better QOL (*p* = 0.042) compared with those who said they had insufficient resources (QOL = 14.77 ± 2.18), similarly the result obtained in the evaluation of QWL, in which teachers who reported having ideal resources (QWL = 3.64 ± 0.38) reported a significantly better QWL (*p* = 0.000) compared with those with acceptable resources (QWL = 3.40 ± 0.38) and insufficient resources (QWL = 3.24 ± 0.49), as well as those who reported having reasonable resources towards those with insufficient resources (Table 4). Other analyses of related to work data relayed in Table 4 were not relevant.

According to Table 5, it was verified that there was a significant moderate positive correlation between QOL and QWL; we could still find significant correlations between QOL and working hours in administrative functions and between QOL and age. In addition, there was a negative correlation between the QWL and the number of students in practical classes.

Through simple linear regression, we could verify that the number of students in practical classes were associated with the QWL, so the higher the number of students in practical classes, the worse the QWL. Furthermore, it was found that the workload for administrative activity was associated with QOL, so that the higher workload the better the QOL (Table 6).

## 4. Discussion

The simultaneous investigation of the quality of life (QOL) and quality of work life (QWL) among university faculty remains relatively uncommon in scholarly research. However, its importance is indisputable given the central role that faculty members play in fostering critical thinking, driving social transformation, and advancing educational development.

This study found no significant differences in QOL and QWL across academic disciplines. Nevertheless, both constructs were substantially influenced by occupational factors, including competitive remuneration, stable statutory employment contracts, the ability to reside in one’s home city, and favorable environmental conditions such as adequate lighting, thermal comfort, reduced noise levels, sufficient teaching materials, and smaller class sizes. Additionally, a strong positive correlation between QOL and QWL was observed, highlighting their intrinsic interdependence.

In line with Koetz et al. [6], faculty reported a moderately satisfactory perception of QOL. Yet, academic teaching is widely recognized as a demanding profession, characterized by long working hours, inadequate rest, and high cognitive demands, which contribute to psychological distress and undermine QOL [15]. The multifaceted roles faculty members assume—including teaching, research, and administrative duties—often reduce time for rest and personal life, leading to exhaustion and compromised well-being.

Faculty continue to face persistent challenges inherent to their profession, notably regarding the work environment and barriers to career progression [16]. Supporting this, research within Brazilian institutions demonstrates that faculty who perceive higher workplace quality of life tend to remain committed to their positions, positively impacting institutional performance [17]. In the context of increasing competition within higher education—particularly among private institutions—public universities confront unique challenges necessitating further investigation [18].

Gender disparities were also evident. Female faculty reported significantly lower QWL perceptions, likely due to the “triple burden” of professional, domestic, and personal responsibilities. This burden limits social engagement, exacerbates physical and emotional strain, and intensifies conflicts between professional and family obligations, often accompanied by guilt [19,20,21].

The environmental domain of QOL received the lowest scores, potentially reflecting external factors such as urban violence, limited professional development opportunities, and suboptimal working conditions in the study setting [22]. These concerns were mirrored in the economic/policy domain of QWL, which encompasses workplace self-esteem, perceived professional value, and career advancement opportunities—all influenced by broader political, social, and economic factors that impact faculty mental health and job satisfaction.

The decrease in QOL in the environmental domain was also reflected in policy/economic sphere of QWL, since in this it expresses self-esteem in the workplace and because what is evaluated in the field and sphere are in part coincident, like the possibility of growth in personal and/or professional level and the social importance of the work. These factors are closely linked to political, economic, and social factors, making them inseparable in the psychological aspect. Moreover, it reflects the lack of opportunities for growth in the analyzed HEI, since most of the teachers had unstable employment, given the employment relationship. Another factor that influenced this sphere was remuneration, since lower-paid teachers reported less satisfaction with QOL, so that they came to understand that their work had such low importance.

In this study, it was verified that those teachers with higher incomes had higher scores in both QOL and QWL. The fact that there are two types of employment ties in the studied institution is cause for discontent and professional dissatisfaction, due to the difference in wages and recognition. Low pay causes the accumulation of jobs, stress, physical exhaustion, and low motivation [21,23]. In addition, the influence of remuneration on QOL and QWL can be explained by the fact that a good income allows housing, keeping healthy habits such as food, leisure, and transport, and it also implies self-esteem, a sense of appreciation, and enjoyment at work [24,25].

Higher income was strongly associated with improved QOL and QWL. Faculty with stable statutory contracts reported greater job security, higher remuneration, and fewer teaching hours, contributing to emotional stability, enhanced life planning, and reduced domestic burden [6]. Conversely, contract faculty lacking job stability experienced increased anxiety and job insecurity, exacerbated by pressures from supervisors, students, and colleagues [10].

Working conditions—specifically lighting, ventilation, temperature, noise, spatial adequacy, and access to teaching materials—exerted significant effects on QWL [9,10,15].

Results of this present study demonstrate the impact on the perception of QWL and QOL by teachers, whose function performance was set in environments with insufficient temperature, lighting, teaching materials, and too much noise. Among the items related to working conditions, thermal comfort and noise are taken by workers as those with the most negative influence on the execution of the work, especially when this is essentially mental/cognitive [26]. Poor thermal comfort and elevated noise levels were frequently identified as detrimental to cognitive performance. Elevated temperatures impair memory and concentration, while excessive noise contributes to irritability, anxiety, sleep disturbances, hypertension, communication difficulties, and job dissatisfaction [27,28]. So, the good quality of working life (QWL) of university professors really improves their well-being [16].

Lighting was another critical determinant. Insufficient illumination adversely affects biological and psychological functioning (QOL dimensions and QWL spheres), while adequate lighting enhances concentration, reduces errors, and improves workplace satisfaction (better QWL) [29]. Poor lighting increases strain on ocular and cervical muscles, demands greater cognitive effort, and leads to fatigue and discomfort [30,31].

Insufficient teaching materials negatively impacted QWL by inducing frustration and helplessness, as faculty invested considerable effort in lesson preparation that was undermined by inadequate classroom resources. Interestingly, faculty engaged in administrative or non-teaching roles reported slightly better QOL, possibly due to reduced exposure to classroom stressors.

Although overall QOL and QWL scores did not significantly differ among different areas, domain-specific differences were noted. Psychological and relational domains consistently scored higher than the environmental domain across fields, except in the exact sciences. This may reflect the younger age and earlier career stage of the exact sciences faculty, who typically teach more courses and hold fewer ancillary roles, potentially limiting institutional support.

However, peculiarities of the areas were exposed when comparing domains, where it was observed that psychological and relational domains were better assessed than the environmental one in all areas except in the exact sciences one; this fact can be explained by the lower average age of these teachers, suggesting professionals just beginning their careers; such an inference is confirmed by the fact that they take a larger number of subjects and do not exercise other paid activities. These characteristics identified may also explain why exact sciences teachers did not realize the slighter economic/political and sociological/relational sphere, as teachers in other areas did [32].

Conversely, the agricultural sciences faculty, often older and more likely to hold doctoral degrees, did not report lower scores in environmental/organizational or economic/policy domains, likely reflecting greater career stability and remuneration [4,8,33].

Among the health sciences faculty, the sociological/relational domain surpassed the biological/physiological domain, likely due to their humanistic training and regular engagement in patient care, fostering empathy and social connectivity.

An Indian study involving 574 university faculty found that 73% were satisfied with their QWL, with male faculty reporting higher satisfaction. Cultural differences in gender roles may account for these divergent findings compared with the current study [34].

In the study by Ramegowda and Kumari [35], they verified, through regression analysis, that working conditions impacted QWL, as was verified in our studies, reaffirming that the current study contains information and findings that are timeless, even to the present day. Similarly, another recent study evidenced that four factors associated with QWL (i.e., management policies, fair pay, work environment, and job design and social space) were identified through exploratory factor analysis (EFA). Subsequently, the relationship between the identified factors of QWL and job performance was modeled through partial least squares structural equation modeling (PLS-SEM). The results inferred that all four factors of QWL had a substantial association with the job performance of the faculty. The study puts forward improvements in the level of existing institutional measures to ensure a superior QWL and the improved performance of faculty in the technical education sector [36].

A survey of 20 Brazilian federal universities revealed that only half implemented formal health promotion and working condition monitoring strategies, underscoring the absence of systematic support for faculty well-being [37]. These findings reinforce the urgent need for institutional initiatives to enhance QWL.

The occupational aspects that impacted QWL and QOL revealed in this study—better remuneration, the statutory agreement, not having to commute from home to act as a professor, better lighting, comfortable temperature, less noise, material resources, and fewer students in class—have the potential to impact professors in recent and important changes in teaching activities, such as teleworking, the incorporation of digital technologies, and artificial intelligence. These factors apply to teachers even when teaching is remote or hybrid and AI are used for dynamics, as they are intrinsic factors to the teaching process. Even during the pandemic, when teachers had to reinvent their methods and adapt their homes to be their workplace, the findings of this study are applicable, because regardless of the workplace, there must be good lighting and cooling, silence (which during the pandemic was more difficult because the family lived together with the worker and their work), the salary was not changed (and in many situations decreased due to the reduction in classes due to student withdrawals); thus, the educators experienced increased stress levels, burnout, and secondary trauma [38].

A recent study conducted in Turkey also verified the impact of occupational factors on QWL. It, like ours, found that noise, temperature, lighting, and physical resources negatively impacted teachers’ QWL. Another recent study, like ours, identified that career satisfaction, job stability, compensation, job location, working conditions, job stress, family–work–life balance, and self-control over work impact QOL. These findings reinforce the contemporary nature of our study and indicate that such findings are multi-cultural [5].

An important study that evaluated the quality of work–life approach found that working conditions and work and total living space indicated intermediate satisfaction, since, with regard to working conditions, the use of technology and the fatigue caused by work were significant indicators [39].

Continuing this line of thought, a survey of 321 teachers obtained that teachers with a higher QWL showed greater psychological empowerment and embraced stronger growth mindsets about their teaching abilities, which enabled them to become more innovative in their teaching approaches [40].

Finally, the strong positive correlation between QOL and QWL observed aligns with prior research [9,41]. Factors such as income, institutional reputation, job satisfaction, physical working conditions, and personal safety directly influence overall quality of life by fostering health, emotional equilibrium, self-esteem, motivation, and a balanced work–life integration.

The fact that the study was conducted at a single institution may be a weakness. However, current studies, including in other countries with cultures different from the one where the study was conducted, corroborate our findings. Thus, the authors understand that the results can be extrapolated to or reflected in other institutions and other countries, since the findings are not influenced by nationality or culture; they are occupational factors that are present in any location and university.

## 5. Conclusions

Based on the findings of this study, it can be concluded that quality of life (QOL) and quality of work life (QWL) are interdependent constructs among higher education faculty. Male professors reported a more favorable perception of both QOL and QWL compared with their female counterparts. Additionally, no significant differences were observed in the QOL and QWL across different academic disciplines, suggesting that the challenges and demands inherent to the teaching profession are shared across fields of knowledge.

The results also highlight that organizational and occupational factors related to the teaching function—such as employment stability, remuneration, working conditions, and access to resources—play a decisive role in shaping both QOL and QWL. These factors are critical for institutional planning and policy development aimed at improving faculty well-being.

Although the study was conducted in a single public higher education institution in Brazil, its findings provide important insights into the broader academic context. Given the scarcity of studies examining the interrelation between QOL and QWL among university faculty, these results may have implications beyond the immediate regional setting, particularly with regard to the confirmed interdependence between QOL and QWL.

Further research is recommended to expand on these findings in other institutional and geographic contexts, allowing for more robust comparisons and the development of targeted strategies to enhance both the professional and personal lives of educators.

## Figures and Tables

**Table 1:** Mean values and comparison of the scores of QOL domains and spheres of QWL (*N* = 284).

	Domains/Spheres	Mean ± SD
**QOL**	Physical	15.84 ± 2.29 ^a^
	Psychological	15.70 ± 2.25 ^a^
	Social relationships	15.63 ± 2.70 ^a^
	Environmental	14.38 ± 1.93
	Overall score QOL	15.20 ± 1.74
**QWL**	Biological/Physiological	3.30 ± 0.48 ^b^
	Psychological/Behavioral	3.78 ± 0.49 ^b,c,d^
	Sociological/Relational	3.46 ± 0.60 ^b,c^
	Economic/Policy	3.07 ± 0.59
	Environmental/Organizational	3.42 ± 0.53 ^b^
	Overall score QWL	3.42 ± 0.41

^a^ Higher compared with the environmental domain; ^b^ higher compared with the economic/policy sphere; ^c^ higher compared with the biological/physiological sphere; ^d^ higher compared with the sociological/relational and environmental/organizational spheres. Analysis by Kruskal–Wallis. Results of comparisons considering *p* < 0.05.

**Table 2:** Categorized sociodemographic data, QOL, and QWL spread over area of knowledge (*N* = 284).

Variables	Health/Biology	Human	Exact	Agricultural	QOL	QWL
	(*N* = 113)	(*N* = 82)	(*N* = 51)	(*N* = 38)	(*N* = 284)	(*N* = 284)
**Sex**						
Male	50 (44.2%)	48 (58.5%)	40 (78.4%)	27 (71.1%)	15.37 ± 1.69	3.47 ± 0.40 ^a^
Female	63 (55.8%)	34 (41.5%)	11 (21.6%)	11 (28.9%)	14.95 ± 1.79	3.35 ± 0.42
**Age**						
20–30	24 (21.2%)	20 (24.4%)	21 (41.2%)	2 (5.3%)	---	---
31–40	65 (57.5%)	24 (29.3%)	14 (27.5%)	11 (28.9%)	---	---
41–50	14 (12.4%)	26 (31.7%)	11 (21.6%)	11 (28.9%)	---	---
More than 51	10 (8.8%)	12 (14.6%)	5 (9.8%)	14 (36.8%)	---	---
**Title**						
Graduation	7 (6.2%)	6 (7.3%)	9 (17.6%)	0 (0%)	15.31 ± 1.73	3.47 ± 0.43
Expert	49 (43.4%)	40 (48.8%)	23 (45.1%)	1 (2.6%)	15.11 ± 1.8	3.37 ± 0.4
Master	49 (43.4%)	32 (39%)	18 (35.3%)	18 (47.4%)	15.16 ± 1.9	3.5 ± 0.55
Ph.D.	8 (7.1%)	4 (4.9%)	1 (2%)	19 (50%)	15.23 ± 0.94	3.58 ± 0.32
**Salaries**						
1–2 salaries	13 (11.5%)	9 (11%)	4 (7.8%)	0 (0%)	15.40 ± 1.74	3.41 ± 0.40
2–3 salaries	13 (11.5%)	20 (24.2%)	6 (11.8%)	4 (10.5%)	14.77 ± 1.48	3.26 ± 0.39
3–5 salaries	32 (28.3%)	22 (26.8%)	26 (51%)	4 (10.5%)	14.95 ± 1.64	3.57 ± 0.43 ^b^
5–10 salaries	34 (30.1%)	25 (30.5%)	13 (%25.5)	8 (21.1%)	15.30 ± 1.75	3.42 ± 0.45
More than 10	21 (18.6%)	6 (7.3%)	2 (3.9%)	22 (57.9%)	15.7 ± 2.03 ^b^	3.39 ± 0.38
**Public student**						
Graduation	113 (100%)	82 (100%)	51 (100%)	38 (100%)	15.16 ± 1.77	3.43 ± 0.41
Expert	13 (11.5%)	9 (11%)	4 (7.8%)	6 (15.8%)	15.47 ± 1.62	3.39 ± 0.45
Stricto sensu	0 (0%)	0 (0%)	0 (0%)	8 (21.1%)	15.28 ± 1.38	3.14 ± 0.43
**Type of contract**						
Statutory	37 (32.7%)	23 (28%)	11 (21.6%)	28 (73.7%)	16.51 ± 1.29 ^c^	3.84 ± 0.12
Non-statutory	68 (60.2%)	58 (70.7%)	38 (74.5%)	7 (18.4%)	15.05 ± 1.54	3.44 ± 0.40
**Works in more than one HEI**						
Yes	12 (10.6%)	4 (4.9%)	5 (9.8%)	2 (5.3%)	14.81 ± 1.75	3.34 ± 0.35
No	101 (89.4%)	78 (95.1%)	46 (90.2%)	36 (94.7%)	15.25 ± 1.74	3.42 ± 0.42
**Traveling to teaching**						
Yes	14 (12.4%)	2 (2.4%)	2 (3.9%)	4 (10.5%)	15.08 ± 1.64	3.27 ± 0.41
No	99 (87.6%)	80 (97.6%)	49 (96.1%)	34 (89.5%)	15.21 ± 1.76	3.43 ± 0.42 ^d^
**Other remunerated activities**						
Yes	71 (62.8%)	54 (65.9%)	18 (35.3%)	6 (15.8%)	15.24 ± 1.76	3.44 ± 0.45
No	42 (37.2%)	28 (24.1%)	33 (64.7%)	32 (84.2%)	15.14 ± 1.74	3.39 ± 0.38
**Conducting ongoing obtain title**						
Yes	33 (29.2%)	23 (28%)	18 (35.3%)	10 (26.3%)	15.18 ± 1.81	3.45 ± 0.41
No	80 (70.8%)	59 (72%)	33 (64.7%)	28 (73.7%)	15.20 ± 1.73	3.41 ± 0.42

^a^ Compared with female; ^b^ compared with 2–3 salaries; ^c^ compared with non-statutory; ^d^ compared with travel. Analysis by Mann–Whitney and Kruskal–Wallis tests. Results of comparisons considering *p* < 0.05. Note: Salaries refers to the minimum wage stipulated by the federal government.

**Table 3:** Mean values and comparison of scores of the domains and spheres in each area of knowledge (*N* = 284).

	Domains/Spheres	Health/Biology	Human	Exact	Agricultural
		(*N* = 113)	(*N* = 82)	(*N* = 51)	(*N* = 38)
**QOL**	Physical	15.77 ± 2.31	15.78 ± 2.27	15.94 ± 2.49	16.06 ± 2.09
	Psychological	15.54 ± 2.39	16.00 ± 2.11	15.28 ± 2.30	16.12 ± 1.99
	Social relationships	15.56 ± 2.84	15.77 ± 2.51	15.32 ± 2.95	15.96 ± 2.41
	Environmental	14.47 ± 1.88 ^a,b^	14.36 ± 1.94 ^a,b^	14.06 ± 2.19 ^a^	14.54 ± 1.67 ^a,b^
	Overall score QOL	15.15 ± 1.84	15.24 ± 1.63	14.98 ± 1.98	15.4 ± 1.55
**QWL**	Biological/Physiological	3.26 ± 0.53 ^e^	3.27 ± 0.46	3.44 ± 0.47	3.29 ± 0.41
	Psychological/Behavioral	3.77 ± 0.56 ^c,f,g^	3.82 ± 0.46 ^c,f,g^	3.74 ± 0.45 ^c,g^	3.74 ± 0.40 ^c,f^
	Sociological/Relational	3.48 ± 0.62 ^d,e^	3.52 ± 0.57 ^e^	3.35 ± 0.61 ^e^	3.26 ± 0.57
	Economic/Policy	3.09 ± 0.60	3.08 ± 0.57	3.09 ± 0.55	2.93 ± 0.65
	Environmental/Organizational	3.43 ± 0.59 ^e^	3.44 ± 0.50 ^e^	3.38 ± 0.51	3.46 ± 0.42 ^e^
	Overall score QWL	3.42 ± 0.47	3.44 ± 0.39	3.45 ± 0.4	3.36 ± 0.31

^a^ Lower compared with the physical domains, ^b^ lower compared with the psychological and social relationships domains, ^c^ higher compared with the biological/physiological and economic/policy spheres, ^d^ higher compared with the biological/physiological sphere, ^e^ higher compared with the economic/policy sphere, ^f^ higher compared with the sociological/relational sphere, ^g^ higher compared with the environmental/organizational sphere. Analysis by Kruskal–Wallis. Results of comparisons considering *p* < 0.05.

**Table 4:** Categorized data related to work QOL and QWL spread over area of knowledge (*N* = 284).

Variables	Health/Biology	Human	Exact	Agricultural	QOL	QWL
	(*N* = 113)	(*N* = 82)	(*N* = 51)	(*N* = 38)	(*N* = 284)	(*N* = 284)
**Number of disciplines**	3.19 ± 1.89	3.06 ± 1.89	4.37 ± 2.03	3.05 ± 1.11	----	----
**Work shift**						
Matutinal	8 (7.1%)	1 (1.2%)	0 (0%)	1 (2.6%)	16.01 ± 2.21	3.72 ± 0.56
Daytime	13 (11.5%)	2 (2.4%)	0 (0%)	30 (78.9%)	15.62 ± 1.55	3.47 ± 0.45
Vespertine	6 (5.3%)	1 (1.2%)	1 (2%)	1 (2.6%)	15.91 ± 1.01	3.44 ± 0.27
Morning and evening	24 (21.2%)	27 (32.9%)	6 (11.8%)	2 (5.3%)	15.08 ± 1.94	3.47 ± 0.49
Afternoon and evening	10 (8.8%)	3 (3.7%)	11 (21.6%)	1 (2.6%)	14.71 ± 1.95	3.43 ± 0.71
Evening	8 (7.1%)	34 (41.5%)	21 (41.2%)	0 (0%)	15.06 ± 1.79	3.41 ± 0.39
Integral	44 (38.9%)	14 (17.1%)	12 (23.5%)	3 (7.9%)	15.38 ± 1.96	3.42 ± 0.34
**Temperature**						
Comfortable	47 (41.6%)	33 (40.2%)	20 (39.2%)	20 (52.6%)	15.35 ± 1.68	3.50 ± 0.4 ^a^
Reasonable	41 (36.3%)	38 (46.3%)	22 (43.1%)	9 (23.7%)	14.96 ± 1.79	3.39 ± 0.43
Uncomfortable	23 (20.4%)	11 (13.4%)	9 (17.6%)	9 (23.7%)	15.34 ± 1.83	3.31 ± 0.4
Unbearable	2 (1.8%)	0 (0%)	0 (0%)	0 (0%)	15.73 ± 1.63	3.02 ± 0.13
**Lighting**						
Enough	73 (64.6%)	55 (67.1%)	35 (68.6%)	32 (84.2%)	15.31 ± 1.65	3.48 ± 0.41 ^b^
Regular	36 (31.9%)	25 (30.5%)	16 (31.4%)	6 (15.8%)	15.06 ± 1.88	3.32 ± 0.41
Inadequate	4 (3.5%)	2 (2.4%)	0 (0%)	0 (0%)	13.91 ± 2.30	3.03 ± 0.35
**Noises**						
None	4 (3.5%)	6 (7.3%)	2 (3.9%)	3 (7.9%)	15.46 ± 1.7 ^g^	3.71 ± 0.39 ^c^
Few	42 (37.2%)	26 (31.7%)	19 (37.3%)	15 (39.5%)	15.37 ± 2.12	3.52 ± 0.41 ^d^
Moderate	57 (50.4%)	42 (51.2%)	24 (47.1%)	20 (52.6%)	14.92 ± 1.70	3.34 ± 0.4
Excessive	10 (8.8%)	8 (9.8%)	6 (11.8%)	0 (0%)	15.27 ± 1.46	3.3 ± 0.4
**Physical space of lectures**						
Ideal	93 (82.3%)	73 (89%)	45 (88.2%)	33 (86.8%)	15.12 ± 1.75	3.33 ± 0.52
No ideal	20 (17.7%)	9 (11%)	6 (11.8%)	5 (13.2%)	15.21 ± 1.75	3.43 ± 0.40
**Physical space of practices**						
Ideal	58 (66.7%)	36 (66.7%)	16 (53.3%)	29 (80.6%)	15.30 ± 1.65	3.36 ± 0.37
Not ideal	29 (33.3%)	18 (33.3%)	14 (46.7%)	7 (19.4%)	15.07 ± 1.85	3.46 ± 0.44
**Physical space of research**						
Ideal	23 (50%)	26 (55.3%)	11 (55%)	19 (61.3%)	15.39 ± 1.60	3.34 ± 0.34
Not ideal	23 (50%)	21 (44.7%)	9 (45%)	12 (38.7%)	15.13 ± 1.46	3.40 ± 0.40
**Resources for teaching**						
Ideal	21 (18.6%)	5 (6.1%)	11 (21.6%)	6 (15.8%)	15.67 ± 1.58 ^h^	3.64 ± 0.38 ^e,f^
Acceptable	74 (65.5%)	67 (81.7%)	30 (58.8%)	19 (50%)	15.18 ± 1.63	3.40 ± 0.38
Inadequate	18 (15.9%)	10 (12.2%)	10 (19.6%)	13 (34.2%)	14.77 ± 2.18	3.24 ± 0.49

^a^ Compared with the uncomfortable; ^b^ compared with the regular and inadequate; ^c^ compared with the moderate and excessive; ^d^ compared with the moderate; ^e^ compared with the inadequate; ^f^ compared with the acceptable; ^g^ compared with the moderate; ^h^ compared with inadequate. Analysis by Mann–Whitney and Kruskal–Wallis tests. Results of comparisons considering *p* < 0.05.

**Table 5:** QOL and QWL correlation with the quantitative variables (*N* = 284).

Variables	QOL		QWL	
	Correlation	*p* Value	Correlation	*p* Value
QOL	----	----	0.564	0.000 *
QWL	0.564	0.000 *	----	----
Age	0.119	0.045 *	−0.012	0.845
Career longevity	0.069	0.251	−0.006	0.926
Number of practical classes	−0.087	0.147	−0.131	0.070
Number of lectures	−0.075	0.206	−0.027	0.651
Workload of stage	−0.016	0.792	0.032	0.588
Workload of research	0.076	0.206	−0.007	0.911
Workload of extension	0.020	0.741	−0.023	0.706
Workload of administrative	0.143	0.016 *	0.058	0.328
Other workloads	0.130	0.115	0.064	0.286
Number of students in practical classes	−0.057	0.335	−0.140	0.018 *
Extra teaching hours	−0.009	0.879	0.075	0.208

* *p* < 0.05; Analysis by Spearman coefficient correlation.

**Table 6:** Simple linear regression analysis to determine association of QOL and QWL with independent variables.

Dependent Variable	Factors	β	*p* Value
QOL	Workload of administrative	0.024	0.023 *
QWL	Number of students in practical classes	−0.39	0.018 *

* *p* < 0.05.

## Data Availability

The data presented in this study are available on request from the corresponding author due to ethical reasons.
